# Acute Kidney Injury in East Africa in an Era of Converging Renal Stressors: A Multiple‐Hit Narrative Review

**DOI:** 10.1155/ijne/4263572

**Published:** 2026-11-06

**Authors:** Abdullahi Abdi Yusuf, Deka Ahmed Abdi, Ayan Mohamed Hassan, Aden Mohamed Hirsi, Iqra Abdirazak Kabayare, Ayan Ahmed Mohomed, Abdihamid Hirsi Abdulle, Khalid Hussein Ulusow

**Affiliations:** ^1^ Faculty of Medicine and Surgery Somali National University Mogadishu Somalia snu.edu.so; ^2^ BENAADIR Research Consultancy and Evaluation Center Mogadishu Somalia; ^3^ Somali Health Evidence and Research Alliance Mogadishu Somalia

**Keywords:** acute kidney injury, East Africa, hemodynamic compromise, infection, multiple-hit injury, nephrotoxicity, volume depletion

## Abstract

**Background:**

Acute kidney injury (AKI) remains an important cause of morbidity and mortality in East Africa, where infectious disease, volume depletion, hemodynamic instability, and nephrotoxic exposure frequently occur in resource‐constrained clinical settings. This review aimed to synthesize regional evidence through a multiple‐hit framework focused on three modifiable renal stressor domains.

**Methods:**

A structured narrative review of PubMed, Embase, and Scopus was conducted for peer‐reviewed human studies published from January 2012 to 18 August 2026 across the 18 Eastern African countries defined by the United Nations M49 framework. Eligible studies examined AKI in relation to volume depletion or hemodynamic compromise, infection or inflammatory illness, and/or nephrotoxic exposure.

**Results:**

Of 902 records identified, 420 duplicates were removed, 482 records were screened, 66 full‐text reports were assessed, and 45 studies were included. Evidence originated from only six countries: Ethiopia, Uganda, Tanzania, Kenya, Somalia/Somaliland, and Rwanda. Across adult, pediatric, neonatal, obstetric, infectious‐disease, emergency, cardiac, and critical‐care populations, AKI was associated with dehydration, hemorrhage, hypotension, shock, malaria, sepsis and other infections, nephrotoxic medications, iodinated contrast, and traditional or herbal medicines. The most consistently documented pattern was cohort‐level clustering of more than one renal stressor domain within the same clinical populations. Individual‐level coexistence was documented in selected studies, whereas detailed temporal sequencing was uncommon. Most studies assessed exposures separately and rarely examined cumulative effects, exposure timing, or statistical interaction.

**Conclusion:**

The currently available evidence from East Africa supports a multiple‐hit perspective as a clinical organizing framework rather than a proven biological or prognostic model. Because the evidence is geographically concentrated, particularly in Ethiopia, Uganda, and Tanzania, these findings should not be interpreted as representative of all East African settings. Recurrent cohort‐level clustering of infectious or inflammatory, hemodynamic, and nephrotoxic stressors was well documented, but patient‐level coexistence and temporal sequence remain incompletely characterized. Prospective multicountry studies with patient‐level temporal data are needed to determine whether the timing, overlap, duration, or combination of these stressors influences AKI severity, recovery, and longer‐term outcomes.

## 1. Introduction

Acute kidney injury (AKI) is a clinical syndrome defined by an abrupt decline in kidney excretory function, and it carries a substantial burden of morbidity and mortality across diverse healthcare settings [1, 2]. A large meta‐analysis of 154 studies involving more than 3.5 million participants reported pooled AKI incidence rates of 21.6% in adults and 33.7% in children, with AKI‐associated mortality of 23.9% and 13.8%, respectively [3]. Beyond the acute episode, AKI is associated with prolonged hospitalization and an increased risk of progression to chronic kidney disease (CKD) and cardiovascular morbidity [1, 2]. The etiology of AKI is heterogeneous, with contributors traditionally classified as prerenal, intrinsic, or postrenal, though most clinical episodes involve overlapping pathophysiological mechanisms [1]. Because many precipitating factors are potentially reversible, identifying modifiable contributors early in the clinical course remains a priority for reducing AKI‐related harm [2].

The epidemiology of AKI in Africa differs from that in high‐income regions, where the condition is predominantly hospital‐acquired and linked to older age and comorbidities [2, 4]. In sub‐Saharan Africa, infections (including malaria, HIV, and diarrheal diseases), nephrotoxic agents, and obstetric complications are commonly reported contributors, and a substantial proportion of AKI is community‐acquired [2, 4]. Reliable incidence statistics for the region remain limited, and reported mortality is substantial, partly reflecting late presentation and constrained access to diagnostic and kidney‐supportive interventions [4]. Within East Africa specifically, a single‐center ICU study in northern Tanzania reported AKI in 55.3% of critically ill adults, with sepsis independently associated with AKI [5]. A separate prospective cohort from northern Tanzania reported in‐hospital mortality of 39.6% among patients with AKI and identified volume depletion and traditional herbal medicine intoxication as independent predictors of death [6]. In Ethiopia, a pediatric ICU case–control study identified sepsis, shock, and nephrotoxic drug exposure as factors significantly associated with AKI [7]. Together, these studies illustrate the heterogeneity of AKI across regional populations and clinical settings, while also showing that several potentially modifiable contributors may be present within the same high‐risk populations [5–7].

Volume depletion or hemodynamic compromise, infection or inflammatory illness, and nephrotoxic exposure are each established contributors to AKI. Volume depletion reduces renal perfusion and, when prolonged, may progress from functional impairment to intrinsic kidney injury [1]. Infection‐associated AKI, particularly in sepsis, involves circulatory, microvascular, inflammatory, mitochondrial, and metabolic disturbances [8]. Malaria‐associated AKI is well documented in African children and is associated with increasing mortality across AKI severity stages [9]. Nephrotoxic medications and other toxic exposures represent an additional potentially modifiable source of kidney injury [10, 11]. These domains are clinically distinct but not mutually exclusive, creating the possibility that more than one renal stressor may be present during the same acute illness [1].

Although multiple renal stressors may be represented within the same clinical setting, interpretation requires a distinction between cohort‐level clustering and documented individual‐level coexistence. In northern Tanzania, volume depletion and traditional herbal medicine intoxication were reported as independent predictors of mortality among patients with AKI [6]. In an Ethiopian pediatric intensive care study, shock, sepsis or severe infection, and nephrotoxic drug exposure were independently associated with AKI within the same clinical population [7]. These findings show that several potentially modifiable renal stressors can cluster within high‐risk populations, but they do not establish that all exposures occurred in the same individual, followed a defined temporal sequence, or interacted biologically. This distinction is central to how the multiple‐hit framework is interpreted in this review.

Existing African and global AKI reviews have addressed epidemiology, individual etiologies, and outcomes in broad terms [2, 4]. Malaria‐associated, sepsis‐associated, and nephrotoxic drug‐associated AKI have also largely been examined within separate disease or exposure frameworks [8–10]. However, it remains unclear how consistently the same potentially modifiable renal stressors recur across different diseases, age groups, and care settings within East Africa, and whether the available evidence demonstrates only population‐level clustering or more direct coexistence within individual clinical episodes. Examining these domains together may therefore reveal cross‐cutting patterns that are less apparent when disease‐specific forms of AKI are considered separately. Such a synthesis may also help identify where current evidence may inform recognition and monitoring and where patient‐level data on timing, overlap, and cumulative exposure are still lacking.

This review aims to synthesize and critically interpret peer‐reviewed evidence on volume depletion or hemodynamic compromise, infection or inflammatory illness, and nephrotoxic exposure as independent and potentially coexisting contributors to AKI in East African populations. Specifically, it examines evidence supporting each exposure domain and evaluates whether multiple renal stressors are reported within the same clinical populations, documented to coexist within individual clinical episodes, or observed at different stages of illness or treatment. It also considers clinically important AKI outcomes, including mortality, kidney replacement therapy requirements, and renal recovery, where data are available, and identifies opportunities for early recognition and prevention. The review further characterizes methodological and geographic gaps that limit interpretation of exposure timing, overlap, and cumulative renal risk. By integrating evidence across these three domains rather than considering them separately, the review seeks to clarify both the clinical relevance and the evidentiary limits of a multiple‐hit perspective on AKI in East Africa.

The principal contribution of this review is therefore the organization of currently available East African evidence according to the level at which multiple renal stressors are documented, namely cohort‐level clustering, patient‐level coexistence, and temporal sequence, rather than the proposal of a new mechanistic model of AKI.

## 2. Methods

### 2.1. Review Design and Scope

This study was conducted as a narrative review using a structured literature search and predefined eligibility criteria. The review focused on three clinically relevant exposure domains associated with AKI: volume depletion or hemodynamic compromise, infection or inflammatory illness, and nephrotoxic exposure. The conduct and reporting of the review were guided by the Scale for the Assessment of Narrative Review Articles (SANRA), while the study‐selection process was documented using a PRISMA 2020 flow diagram to improve transparency. The geographic scope comprised the 18 sovereign countries classified within Eastern Africa according to the United Nations M49 geographic framework. Studies published from January 2012 to 18 August 2026 were considered eligible. The starting year of 2012 was selected to correspond with the publication of the kidney disease: Improving Global Outcomes (KDIGO) AKI guideline and improving comparability across contemporary AKI definitions.

### 2.2. Information Sources and Search Strategy

PubMed, Embase, and Scopus were systematically searched for eligible studies published between January 2012 and 18 August 2026. Search strategies combined terms related to AKI with terms representing the three predefined exposure domains: volume depletion or hemodynamic compromise, infection or inflammatory illness, and nephrotoxic exposure, together with the names of the 18 eligible Eastern African countries. Database‐specific controlled vocabulary and free‐text terms were adapted to the indexing requirements of each database. The complete search strategies for all three databases are provided in Supporting Table S1.

### 2.3. Eligibility Criteria

Peer‐reviewed original human studies were eligible if they were conducted in one of the predefined Eastern African countries, were published between January 2012 and 18 August 2026, and examined AKI as an explicit and clinically meaningful outcome. Studies were required to report or examine a direct and relevant relationship between AKI and at least one of the three predefined exposure domains: volume depletion or hemodynamic compromise, infection or inflammatory illness, or nephrotoxic exposure. Studies were not required to assess all three domains. Eligible designs included prospective and retrospective cohort studies, cross‐sectional studies, case–control studies, clinical trials, and informative case series with at least five participants.

Reviews, editorials, commentaries, protocols, conference abstracts, preprints, single case reports, and case series with fewer than five participants were excluded. Studies were also excluded when AKI was incidental or not analytically distinct, when the reported determinants of AKI fell outside the predefined exposure domains, or when the study focused primarily on consequences of established AKI without providing relevant exposure‐to‐AKI evidence. Secondary analyses of overlapping cohorts were excluded when they did not contribute meaningful new evidence relevant to the review framework.

### 2.4. Study Selection

Search results from all databases were combined, and duplicate records were identified and removed before screening. Two reviewers independently screened titles and abstracts against the predefined eligibility criteria. Potentially relevant reports were retrieved for full‐text assessment, which was also performed independently by the two reviewers using the same eligibility framework. Reasons for full‐text exclusion were documented. Any disagreements regarding eligibility were resolved through discussion. The complete study‐selection process is summarized in Figure 1.

**FIGURE 1:**
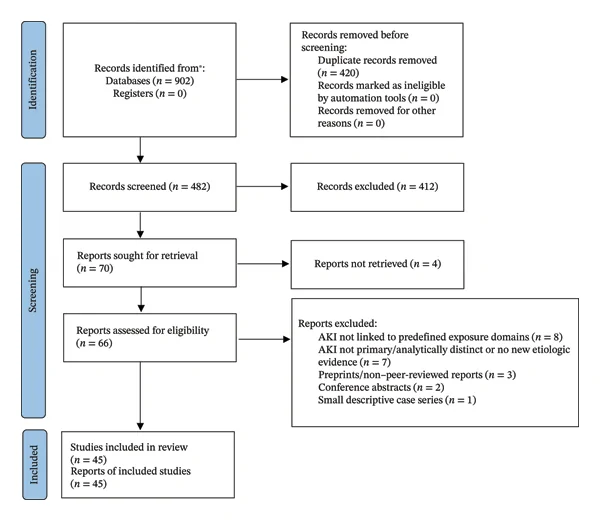
PRISMA 2020 flow diagram of study identification, screening, eligibility assessment, and inclusion in the narrative review.

### 2.5. Evidence Organization and Synthesis

Included studies were organized according to country, population, clinical setting, AKI definition, relevant exposure domain, and key clinical outcomes. Findings were grouped into three predefined domains: volume depletion or hemodynamic compromise, infection or inflammatory illness, and nephrotoxic exposure. Studies reporting more than one domain were further examined according to the level of evidence available: whether multiple stressors were reported within the same clinical population, explicitly documented within the same individual clinical episode, or reported at different stages of illness or treatment when temporal information was available. These categories were interpreted separately, and cohort‐level clustering was not considered evidence of individual‐level coexistence or sequential accumulation. Sepsis, suspected sepsis, severe infection, and other infectious syndromes were classified according to the terminology and diagnostic criteria reported in the original studies. We did not infer sepsis from other infection‐related labels or retrospectively harmonize heterogeneous case definitions across populations or settings. Because of heterogeneity in study populations, settings, AKI definitions, exposures, and outcomes, the evidence was synthesized narratively rather than quantitatively pooled. Because the three renal stressor domains were defined a priori and incorporated into the search and eligibility criteria, their recurrence among the included studies should not be interpreted as evidence that these domains emerged independently from an unrestricted assessment of all possible contributors to AKI in East Africa. Rather, the review evaluates patterns of cohort‐level clustering, patient‐level coexistence, and temporal sequence within these prespecified domains. Contributors outside these domains were not systematically captured; therefore, the framework should not be considered exhaustive or interpreted as establishing the relative importance of these domains compared with other contributors to AKI.

### 2.6. Methodological Appraisal

The methodological strengths and limitations of the included studies were considered during interpretation of the evidence. Particular attention was given to study design, sample size, AKI definition, exposure ascertainment, adjustment for confounding, and the possibility of residual confounding by disease severity or treatment indication when interpreting associations with therapeutic exposures, as well as completeness of outcome reporting. Treatment‐related associations were therefore interpreted as observational associations unless the study design and analysis supported a stronger inference. No formal study‐level risk‐of‐bias score was used because the review was designed as a narrative review rather than a systematic review. Accordingly, comparisons across studies reflect the consistency and level of documentation in the available literature rather than a formally graded hierarchy of evidence quality or certainty.

## 3. Results

### 3.1. Study Selection and Characteristics

The database searches identified 902 records. After removal of 420 duplicates, 482 records underwent title and abstract screening. Seventy reports were sought for full‐text retrieval, of which four could not be obtained. Sixty‐six full‐text reports were assessed for eligibility; 21 were excluded, resulting in 45 studies included in the narrative review. The study‐selection process, including reasons for full‐text exclusion, is presented in Figure 1.

The included evidence originated from only 6 of the 18 predefined Eastern African countries, indicating substantial geographic concentration of the regional literature. Ethiopia contributed the largest number of studies (*n* = 14), followed by Uganda (*n* = 11), Tanzania (*n* = 10), Kenya (*n* = 4), Somalia/Somaliland (*n* = 4), and Rwanda (*n *= 2); no eligible studies were identified from the remaining 12 countries. The studies encompassed adults, children, neonates, and pregnant or postpartum populations across emergency, inpatient, intensive‐care, infectious‐disease, obstetric, and cardiac settings. Observational designs predominated, and KDIGO or KDIGO‐derived criteria were most commonly used to define AKI, although AKIN and other creatinine‐based definitions were also reported.

### 3.2. Volume Depletion and Hemodynamic Compromise

Hemodynamic and volume‐related factors were independently associated with AKI across pediatric, neonatal, trauma, and adult critical‐care cohorts [7, 12–15]. Among Ugandan children hospitalized with sickle cell anemia, hypovolemia was independently associated with AKI (aOR 2.98, 95% CI 1.08–8.23) [12]. Similarly, dehydration was independently associated with AKI among preterm neonates in Ethiopia (AOR 2.30, 95% CI 1.29–4.09) [13], while hypovolemic shock was associated with AKI among critically ill Ethiopian children (aOR 3.88) [7]. Among adults with traumatic brain injury, hypotension was associated with more than a threefold higher hazard of AKI (AHR 3.45, 95% CI 1.71–6.96) [14]. In an Ethiopian medical intensive care unit, negative fluid balance (AHR 2.00) and vasopressor use (AHR 1.72) were independently associated with a higher hazard of AKI [15].

Volume‐related factors were also reported in infection‐associated cohorts. Among adults hospitalized with malaria in southwest Ethiopia, dehydration was independently associated with AKI (aOR 1.74, 95% CI 1.01–3.02) [16]. In Rwanda, AKI was documented in approximately 16% of patients with severe malaria, and dehydration, hypovolemia, profuse sweating, vomiting, and diarrhea were associated with AKI in unadjusted comparisons [17]. Among Ugandan adults with sepsis, a postural systolic blood pressure decrease greater than 9 mmHg was associated with AKI in bivariate analysis, but this association was not statistically significant after age adjustment [18].

Obstetric studies identified hemorrhage and volume depletion as important components of pregnancy‐related AKI, but these occurred within a broader pattern of pregnancy‐specific and infectious conditions. In Tigray, prerenal AKI attributed to hemorrhage or dehydration was reported in 40.6% of women with pregnancy‐related AKI, while hypertensive disorders of pregnancy were reported in 75.4%, including preeclampsia in 71.1%; sepsis and HELLP syndrome were reported in 49.2% and 26.7%, respectively [19]. In a Kenyan obstetric critical‐care cohort, hypertensive disorders of pregnancy were reported in 64.1% of cases, obstetric hemorrhage in 38.4%, and sepsis in 36.5%; HELLP syndrome was documented in 32.5% [20]. Thus, hemorrhagic or volume‐related compromise represented one important obstetric pathway, but it should not be interpreted in isolation from hypertensive disorders and infection in these pregnancy‐related AKI cohorts.

### 3.3. Infection‐Associated Acute Kidney Injury

Infection represented a major clinical context for AKI across the included East African studies, with evidence spanning malaria, sepsis and other acute infections, neonatal infections, tuberculosis, COVID‐19, and visceral leishmaniasis. Malaria contributed a substantial portion of this evidence. In a prospective Ugandan cohort of children hospitalized with acute febrile illness, AKI occurred in 46.7% of children with malaria and 55.0% of those with nonmalarial febrile illness; circulating markers of immune and endothelial activation were associated with severe AKI, and delayed capillary refill was associated with severe AKI among children with malaria [21]. Among 350 Ugandan children with severe malaria at Kiryandongo General Hospital, AKI occurred in 47.7%, and a history of NSAID exposure was independently associated with AKI (aOR 5.6, 95% CI 2.34–13.22) [22]. In a separate prospective cohort of 598 Ugandan children with severe malaria, AKI occurred in 45.3%; reported herbal medicine use, shock, and vomiting were associated with AKI after adjustment for age, sex, and study site [23]. Across these malaria cohorts, hemodynamic features, gastrointestinal symptoms, and medication or herbal exposures were reported in association with AKI, but these cohort‐level findings do not establish that the different stressors coexisted within the same individual child.

Infection‐related associations with AKI were reported across adult, pediatric, and neonatal populations, although the terminology and case definitions varied between studies. In northern Tanzania, AKI occurred in 55.3% of critically ill adults, and sepsis was independently associated with AKI (OR 3.81, 95% CI 1.21–11.99) [5]. Among critically ill children in Ethiopia, “sepsis or infection” was independently associated with AKI (aOR 3.36, 95% CI 1.42–7.99) [7]. In a multicenter Ethiopian neonatal cohort, AKI occurred in 20.2%, and both sepsis (AHR 2.59, 95% CI 1.21–5.56) and gentamicin exposure (AHR 1.74, 95% CI 1.03–2.94) were independently associated with AKI [24]. Similarly, among critically ill neonates in Tanzania, AKI occurred in 31.5%, with neonatal sepsis, severe pneumonia, and gentamicin exposure independently associated with AKI [25]. In Kenya, AKI occurred in 36.1% of neonates admitted with suspected sepsis and was more common among those with suspected late‐onset sepsis [26]. These studies demonstrate recurrent cohort‐level associations between infection‐related conditions and AKI; in some neonatal cohorts, infection‐related diagnoses and nephrotoxic treatment exposures were both represented, but the available analyses do not establish that these stressors coexisted or occurred sequentially in the same individual neonate.

Infection‐associated AKI was also reported in COVID‐19, tuberculosis, and visceral leishmaniasis. In hospitalized COVID‐19 cohorts, AKI was reported in 21.6% of patients in Kenya, 42% of patients with severe COVID‐19 pneumonia in Ethiopia, and 12.7% of patients in Somalia [27–29]. In Uganda, AKI was identified in 33.3% of adults with recently diagnosed drug‐susceptible tuberculosis during the early postdiagnosis period [30]. Because treatment had already begun in some participants and pre‐enrollment creatinine measurements were unavailable, the study does not establish whether tuberculosis itself or treatment was the direct cause of AKI. In Ethiopian patients with visceral leishmaniasis, 30 patients had AKI at admission before treatment, whereas 22 patients with no AKI at admission developed AKI during treatment; concomitant infections and HIV co‐infection were independently associated with AKI [31]. These findings show that AKI can be detected at different stages of infectious illness and treatment across patients, but they do not establish repeated sequential renal injury within the same individual.

### 3.4. Nephrotoxic Exposure

Potentially nephrotoxic exposures were associated with AKI across adult, pediatric, and neonatal settings. In Ethiopia, hospital‐acquired AKI among non‐critical medical inpatients was independently associated with vancomycin and proton pump inhibitor use, showing that medication‐associated AKI was also reported outside intensive‐care populations [32]. Aminoglycoside exposure was particularly prominent in neonatal and pediatric studies. In Kenya, nephrotoxicity occurred in 10.3% of children receiving gentamicin or amikacin, with neonates and low‐birth‐weight neonates particularly vulnerable [33]. Gentamicin was also independently associated with AKI among neonates in Ethiopia and Tanzania [24, 25], while a Tanzanian study of premature and low‐birth‐weight neonates found that exposure to two nephrotoxic drugs was associated with more than twice the prevalence of AKI [34]. NSAID exposure provided another recurring signal: Prior NSAID use was independently associated with AKI among Ugandan children with severe malaria [22], and frequent NSAID use was independently associated with AKI among hospitalized children with sickle cell disease [35].

Iodinated contrast represented a separate iatrogenic exposure, particularly in Tanzanian cardiac populations. Contrast‐induced nephropathy was reported in 19% of patients undergoing coronary angiography or intervention in one study [36], while another PCI cohort reported AKI in 9.7%, with no statistically significant factors associated with AKI, including contrast volume [37]. A more recent prospective single‐center study involving 187 adults undergoing elective cardiac catheterization reported a markedly higher incidence of 53.5% (100/187), classified by the authors as contrast‐induced nephropathy [38]. This unusually high estimate warrants cautious interpretation. The study applied a serum creatinine increase of at least 0.3 mg/dL within 48 h or 1.5 times baseline within 7 days, a more sensitive definition than that used in some earlier Tanzanian studies. Advanced age, hypertension, and diabetes mellitus were associated with the reported outcome, and 38% of affected patients had pre‐existing CKD. At 3 months, the authors reported new‐onset CKD in 38% and CKD progression in 21% of patients who developed contrast‐associated kidney injury. However, the single‐center observational design, high burden of comorbidities, and absence of a noncontrast comparator limit causal attribution. Postprocedure increases in serum creatinine cannot be attributed exclusively to iodinated contrast, and confounding by underlying renal vulnerability, patient characteristics, or procedural factors cannot be excluded. We retained this study because it met the predefined eligibility criteria and provides relevant regional evidence of kidney dysfunction temporally associated with cardiac catheterization. Nevertheless, its 53.5% estimate should be regarded as context‐specific rather than representative of Tanzania or East Africa, and the three Tanzanian estimates should not be directly compared because of differences in study design, patient characteristics, procedures, and AKI definitions.

Traditional medicines and treatment‐related exposures further broadened the nephrotoxic profile. Among critically ill adults in Tanzania, local herbal medicine use was independently associated with AKI (OR 4.68, 95% CI 1.53–14.33) [39], while herbal medication exposure was also associated with AKI in Ugandan children hospitalized with severe malaria [23]. In Ethiopian patients with visceral leishmaniasis, drug‐induced injury was the presumed cause in 11 of 22 patients who developed AKI after admission, with these cases attributed to sodium stibogluconate‐based therapy [31]. Together, these studies identify traditional medicines and therapeutic drug exposure as additional nephrotoxic contexts within the regional AKI literature.

### 3.5. Convergence of Multiple Renal Stressors

The synthesis identified recurrent clustering of the three renal stressor domains across otherwise distinct East African populations and clinical settings. The most consistently documented pattern was at the cohort level, where more than one domain was associated with AKI within the same clinical population. Selected studies provided evidence of individual‐level coexistence, while temporal information showing that exposures occurred at different stages of illness or treatment was less consistently available. Accordingly, the evidence supports a spectrum ranging from population‐level clustering to more limited patient‐level coexistence and temporal observations, but does not establish a consistent sequence of accumulating renal insults or quantify their joint effects.

Several studies represented all three predefined stressor domains within the same clinical population. In Tanzanian adults with upper gastrointestinal bleeding, rebleeding during admission, hepatitis B infection, and nephrotoxic medication use were independently associated with AKI [40]. In Ethiopian patients with visceral leishmaniasis, dehydration was the main reported etiology of AKI at admission, whereas presumed drug‐induced AKI accounted for 11 of 22 cases that developed after admission [31]. These findings show that different renal stressors were represented at different stages across the cohort, but they do not demonstrate sequential injury in the same individual patient. A pediatric intensive‐care study from Ethiopia showed a similar risk architecture from another perspective, with shock, sepsis or infection, and nephrotoxic drug exposure each independently associated with AKI [7]. Together, these studies demonstrate recurrent cohort‐level clustering across the three domains, while patient‐level coexistence and temporal sequence remain less clearly established.

Treatment‐related exposures were also represented in infection‐dominated clinical settings. Among Ugandan children with severe malaria, shock, vomiting, and prior herbal medicine use were each associated with AKI after adjustment for age, sex, and study site [23]. In critically ill Tanzanian adults, sepsis and local herbal medicine use were independently associated with AKI [39]. More direct individual‐level coexistence was documented among Kenyan neonates receiving aminoglycoside therapy, where those treated for sepsis had a higher risk of nephrotoxicity than neonates treated for other illnesses [33], while an Ethiopian neonatal cohort identified both sepsis and gentamicin exposure as independent AKI predictors [24]. These findings show that infection and potentially nephrotoxic exposures can occur within the same treatment context, but they do not consistently establish their temporal order or isolate treatment‐related kidney injury from the severity of the underlying illness or treatment indication.

A further recurring pattern involved infection and volume or circulatory compromise within the same high‐risk populations. In Tanzanian pregnancy‐related AKI, postpartum hemorrhage and sepsis were both reported as important contributors [41], while the Somaliland cohort similarly included postpartum hemorrhage and puerperal sepsis among the major etiologies [42]. These findings represent cohort‐level clustering rather than documented coexistence in individual women. In contrast, the Somali postpartum AKI study explicitly documented one patient with both sepsis and hemorrhagic shock, providing direct evidence of individual‐level coexistence [43]. Among Ugandan children with acute malnutrition, both diarrhea with vomiting and sepsis were independently associated with AKI [44], while among children with sickle cell anemia, hypovolemia and infection were also independently associated with AKI [12]. Overall, infection and impaired perfusion repeatedly appeared within the same clinical populations, although direct documentation of both stressors in the same individual was uncommon.

Across these studies, exposures were usually analyzed as separate predictors or etiologies, while interaction terms, cumulative exposure measures, and time‐varying analyses were largely absent. The available evidence therefore does not establish additive or synergistic effects between stressors. The multiple‐hit framework should accordingly be interpreted as a clinical organizing lens rather than a demonstrated biological or sequential mechanism. The principal stressor domains and supporting evidence are summarized in Table 1.

**TABLE 1:** Principal renal stressor domains and patterns of convergence associated with acute kidney injury in East Africa.

Renal stressor domain	Where observed	Evidence pattern	What the evidence supports	Representative studies
Volume depletion and hemodynamic compromise	Severe malaria, critical illness, trauma, obstetric AKI, neonatal and pediatric illness	Mainly cohort‐level association with AKI; often reported in populations where infection or other systemic stressors were also present	Hemodynamic compromise is a recurrent modifiable AKI‐associated domain, but its joint effect with other stressors has rarely been quantified	[7, 13–15, 17, 19, 20]
Infection and inflammatory illness	Malaria, sepsis or severe infection, neonatal infection, tuberculosis, COVID‐19, visceral leishmaniasis	Predominantly cohort‐level clustering; selected studies documented patient‐level coexistence with other stressors	Infection is a major AKI‐associated context, but the timing and contribution of accompanying hemodynamic or treatment‐related exposures are usually uncertain	[5, 21, 31, 33, 43]
Nephrotoxic exposure	Aminoglycoside‐treated neonates and children, adult medical inpatients, cardiac catheterization, severe infectious illness	Drug‐ or exposure‐related associations with AKI; individual‐level coexistence documented in selected settings, but temporal attribution often limited	Nephrotoxic exposures are potentially modifiable, although observational findings may be influenced by illness severity and treatment indication	[24, 32, 33, 36, 39]
Converging or multiple renal stressors	Visceral leishmaniasis, severe malaria, critical illness, upper gastrointestinal bleeding, obstetric AKI, neonatal infection	Cohort‐level clustering most consistently documented; patient‐level coexistence less common; detailed temporal sequencing uncommon	Supports a clinical organizing framework for AKI risk assessment but does not establish a consistent sequential pathway, additive effect, or biological synergy	[7, 31, 33, 40, 43]

Taken together, these patterns show that the three renal stressor domains recur within the same high‐risk clinical settings, with cohort‐level clustering being the most consistently documented pattern and more limited evidence available for individual‐level coexistence or temporal sequence. This evidence forms the basis of the conceptual multiple‐hit framework presented in Figure 2.

**FIGURE 2:**
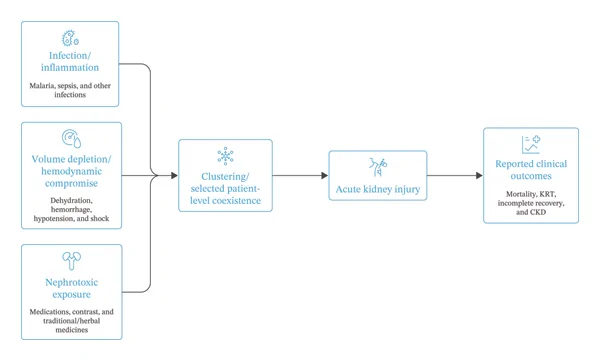
Conceptual multiple‐hit framework for acute kidney injury in East Africa.

Figure 2 illustrates how infection or inflammation, volume depletion or hemodynamic compromise, and nephrotoxic exposure may occur independently or cluster within the same clinical population, with selected evidence of individual‐level coexistence, before being associated with AKI and its reported clinical outcomes. Potential sequential exposure during illness or treatment is conceptually plausible but was not established by most included studies. The arrows represent conceptual associations used to organize the reviewed evidence and do not imply causality or a fixed temporal sequence.

### 3.6. Clinical Outcomes

AKI was associated with clinically important short‐term outcomes, although the magnitude of mortality varied across populations and settings. Among Ugandan children hospitalized with gastroenteritis, malaria, or pneumonia, mortality was 25% in those with AKI compared with 9.9% among children without kidney injury, and AKI remained independently associated with in‐hospital mortality (aOR 3.5, 95% CI 2.2–5.5) [45]. In a separate Ugandan cohort of children hospitalized with acute febrile illness, severe AKI was associated with increased in‐hospital mortality after adjustment for age, sex, and disease severity (RR 2.17, 95% CI 1.01–4.64) [46].

Across the included studies, AKI was associated with incomplete recovery, dialysis requirements, and longer‐term morbidity. An early pediatric series from Somalia reported complete recovery in 61.5% of children, while 20.5% had partial recovery and 17.9% died [47]. Among Ethiopian emergency‐department patients with AKI, 23.4% received hemodialysis, 50.9% were admitted to a ward or intensive care unit, and 9.9% died in the emergency department [48]. Longer‐term consequences were particularly evident in Ugandan children surviving severe malaria, where AKI was associated with subsequent CKD and impaired cognitive outcomes [49]. In an Ethiopian prospective cohort, 61.2% had persistent AKI and 85% experienced prolonged hospitalization [50], while mortality was 29.1% among 151 patients with dialysis‐requiring AKI [51].

Outcome reporting also revealed substantial gaps in assessment of renal recovery. In the multicenter Rwandan pediatric cohort, 76.3% of children presented with KDIGO stage 3 AKI, and overall mortality was 9.6%; hypovolemic shock (OR 8.73, 95% CI 2.13–35.76) and severe dehydration (OR 5.29, 95% CI 1.57–17.86) were independently associated with mortality [52]. Final creatinine measurement before discharge was documented in only 27.5% of patients before and 33.3% after the educational intervention, while renal recovery status was not assessed in 74.4% of cases. Taken together, the included outcome studies demonstrate substantial mortality, dialysis requirements, incomplete recovery, persistent kidney dysfunction, and longer‐term kidney morbidity after AKI across several East African populations.

These outcome data primarily describe the prognosis and clinical burden of AKI itself. Most included studies did not stratify mortality, kidney replacement therapy, renal recovery, or longer‐term outcomes according to the number, combination, or temporal sequence of renal stressors. Accordingly, this section provides clinical context for the consequences of AKI but does not establish that exposure to multiple renal stressors leads to worse outcomes than exposure to a single stressor.

## 4. Discussion

The principal contribution of this review is the structured organization of East African AKI evidence according to whether multiple renal stressors are documented at the cohort level, within individual patients, or in temporal sequence. The review does not propose a new mechanistic model of AKI; rather, it clarifies the level of evidence supporting the multiple‐hit concept across diverse diseases, age groups, and care settings. Cohort‐level clustering of these domains was more consistently documented than direct coexistence within individual patients, while detailed temporal sequencing was uncommon. This distinction limits how far the multiple‐hit concept can be taken mechanistically or prognostically. The included studies do not establish that the number or order of stressors independently determines AKI severity, nor that combined exposure produces additive or synergistic injury. Similarly, the outcome literature summarized in this review largely reflects outcomes associated with AKI itself rather than comparative outcomes according to stressor burden or specific stressor combinations. The clinical value of the framework is therefore more modest but potentially useful: When one dominant cause of AKI is apparent, it prompts consideration of additional, potentially reversible contributors that may otherwise be overlooked. In this sense, the framework is best viewed as an organizing approach to clinical assessment rather than as a new biological model, risk score, or causal pathway.

The present synthesis is best viewed as complementary to, rather than a replacement for, existing African and tropical AKI reviews. Kahindo et al. described the broad epidemiology and etiologic profile of adult AKI across sub‐Saharan Africa, including infection, nephrotoxins, and obstetric and surgical causes [4]. Batte et al. examined malaria‐associated AKI in African children in greater disease‐specific depth, including hypovolemia, inflammatory and endothelial injury, nephrotoxic exposures, outcomes, and management challenges [9]. Kusirisin et al. considered tropical AKI across infections, toxins, herbal and environmental exposures [53], whereas Shankar et al. focused on infection‐associated AKI and highlighted pathways such as volume depletion, hypotension, immune‐mediated injury, and multiorgan dysfunction [54]. The added value of the present review is therefore not the recognition that these mechanisms can coexist. Rather, it is the examination of East African primary studies across different diseases, age groups, and care settings to determine how consistently the same modifiable stressors recur and, importantly, the extent to which the available evidence documents cohort‐level clustering, individual‐level coexistence, and temporal sequence. This regional comparison reveals that clustering is considerably better documented than patient‐level overlap or sequencing, a distinction that broader etiologic or disease‐specific reviews were not designed to evaluate.

Disease‐specific evidence also shows why the three‐domain framework should not be interpreted as a treatment algorithm. Malaria‐associated AKI may involve inflammatory and endothelial injury, hemolysis, hypovolemia, and impaired renal perfusion [9], while sepsis‐associated AKI includes microcirculatory, inflammatory, and metabolic disturbances that cannot be reduced to hypoperfusion alone [55]. Drug‐associated AKI is similarly heterogeneous, with mechanisms varying across tubular toxicity, interstitial injury, altered renal hemodynamics, and other agent‐specific effects [56], while traditional medicines introduce additional uncertainty because their patterns of use and kidney‐related benefits and harms remain incompletely characterized [57]. Obstetric AKI exposes another limitation of a simple three‐domain classification: hemorrhage and infection are important, but the East African pregnancy cohorts also identified hypertensive disorders, preeclampsia, and HELLP syndrome as major clinical contexts [19, 20], consistent with the broader African obstetric literature [58]. The practical implication is that identifying a stressor domain should prompt further assessment rather than dictate a uniform intervention. The framework may help clinicians recognize potentially modifiable contributors, but the appropriate response still depends on the underlying disease, physiological state, treatment indication, and competing risks.

The timing of renal stressors may be clinically relevant, but the current East African evidence does not permit firm conclusions about whether simultaneous or sequential exposure changes prognosis. The distinction between exposure timing and AKI detection is particularly important. In the Ethiopian visceral‐leishmaniasis cohort, 30 patients had AKI at admission before treatment, whereas 22 different patients with initially normal kidney function developed AKI during treatment [31]. This demonstrates that AKI may be detected at different stages of illness and treatment across a cohort, but it does not establish repeated injury or a defined sequence of stressors within the same patient. Similar limitations apply to neonatal studies in which infection‐related diagnoses and gentamicin exposure were both associated with AKI [24, 25], and to pregnancy‐related cohorts in which hemorrhage, sepsis, hypertensive disorders, preeclampsia, and HELLP syndrome were reported [19, 20]. These studies rarely documented the duration of each exposure, the extent of overlap, or whether kidney function recovered between potential insults. Consequently, it is not yet possible to determine whether two concurrent stressors carry the same risk as two separated in time or whether incomplete recovery from an earlier insult increases susceptibility to a later one. Clinically, the value of considering timing is therefore not that a particular sequence has been shown to predict outcome, but that changes in a patient’s condition or treatment may introduce new renal risks that warrant reassessment during the course of illness.

The main clinical challenge is therefore not simply to identify several renal stressors but to manage potentially competing priorities without assuming that every associated exposure is avoidable or equally causal. In infection‐associated AKI, antimicrobial treatment may be essential even when the selected agent has nephrotoxic potential; neonatal and pediatric studies in this review illustrate this tension particularly clearly for aminoglycosides [24, 25, 33]. Similarly, evidence of dehydration, hypotension, or shock should prompt careful assessment of renal perfusion, but it should not imply that all AKI in severe infection is explained by volume depletion or will respond to the same hemodynamic intervention [55]. Potentially nephrotoxic medications or contrast should therefore be evaluated in the context of indication, alternatives, dose, duration, and the patient’s evolving kidney function rather than treated as uniformly preventable exposures [56]. This is also why treatment‐related associations in observational studies require caution: patients receiving aminoglycosides, antileishmanial therapy, vasopressors, or other intensive treatments may have been sicker at baseline, creating residual confounding by disease severity or treatment indication. The practical role of the three‐domain framework is therefore to support repeated reassessment of modifiable contributors while preserving necessary treatment, rather than to assign a single cause or prescribe a fixed bundle of interventions.

Despite these uncertainties, the synthesis identifies several pragmatic priorities that do not require precise attribution of the relative contribution of each stressor. Renal risk should be reassessed when the clinical condition or treatment changes rather than considered fixed at admission, particularly because AKI was identified both before treatment and during treatment in visceral leishmaniasis [31], and potentially nephrotoxic therapies were associated with AKI in neonatal and pediatric populations [24, 25, 33]. Medication review should distinguish exposures that are avoidable from those that are clinically necessary; when a potentially nephrotoxic treatment is required, the emphasis shifts toward appropriate dosing, limiting additional avoidable nephrotoxins, and monitoring kidney function rather than automatic withdrawal of therapy. Assessment should also extend beyond recognition of the acute episode. In the Rwandan pediatric cohort, final creatinine measurement before discharge was documented in only 27.5% of patients before and 33.3% after the educational intervention, while renal recovery status was not assessed in 74.4% [52], highlighting a substantial gap in determining whether kidney function had recovered. Structured approaches to AKI recognition and management have been shown to be feasible in low‐resource settings through the ISN 0by25 initiative [59], although this does not establish that the three‐domain framework proposed here improves clinical outcomes. These measures should therefore be regarded as practical assessment and monitoring priorities rather than a validated AKI prevention bundle.

Pregnancy‐related AKI illustrates both the usefulness and the limits of the three‐domain framework. In the Tigray cohort, hypertensive disorders of pregnancy were reported in 75.4% of women with pregnancy‐related AKI, including preeclampsia in 71.1%, while hemorrhage or dehydration, sepsis, and HELLP syndrome were also represented [19]. A Kenyan obstetric critical‐care cohort similarly reported hypertensive disorders, hemorrhage, sepsis, and HELLP syndrome within the same AKI population [20]. These findings show that hemodynamic compromise and infection are important components of obstetric AKI, but pregnancy‐specific disorders cannot be fully subsumed under those categories. Pregnancy‐related AKI studies from Tanzania and Somaliland also identified hemorrhage and sepsis as important etiologies [41, 42], whereas the Somali postpartum study documented at least one patient with both sepsis and hemorrhagic shock [43]. However, the available studies do not consistently establish whether hypertensive disease, hemorrhage, infection, or treatment occurred sequentially within individual patients, nor do they adequately characterize renal recovery between events. The three‐domain framework should therefore complement, rather than replace, pregnancy‐specific assessment. In obstetric AKI, particular attention remains necessary to hypertensive disorders, preeclampsia, HELLP syndrome, hemorrhage, and infection, while the relative timing and interaction of these conditions remain important evidence gaps. These findings apply specifically to pregnant and postpartum populations and should not be generalized to women outside the obstetric setting.

The geographic and methodological concentration of the evidence substantially limits how confidently these patterns can be considered representative of East Africa as a whole. Only six of the 18 predefined countries contributed eligible studies, and 35 of 45 studies originated from Ethiopia, Uganda, and Tanzania. The apparent prominence of particular stressors may therefore reflect, at least in part, the diseases most often studied, referral‐hospital case mix, access to serum creatinine testing, and local research capacity rather than a uniform regional epidemiology. The same limitation applies to population comparisons: the included studies involved neonates, children, adults, and pregnant or postpartum patients, but few were designed to compare the relative importance, timing, or interaction of renal stressors between these groups. Similarly, the available studies did not provide sufficient sex‐stratified data to determine whether the distribution, timing, or effects of these renal stressors differ between male and female patients outside pregnancy‐related settings. Future studies should therefore prioritize prospective multicountry designs with standardized AKI definitions, explicit baseline‐kidney‐function assessment, repeated creatinine and urine‐output measurements, and patient‐level recording of exposure onset, duration, overlap, and recovery between events. Such data would allow investigators to determine whether the number, combination, or sequence of stressors is independently associated with AKI severity, kidney replacement therapy, renal recovery, mortality, acute kidney disease, or later CKD, rather than assuming that these relationships exist.

This review has several strengths, including a structured search of three major databases, a predefined 18‐country geographic scope, a contemporary evidence window, and inclusion of 45 primary studies spanning adult, pediatric, neonatal, obstetric, infectious‐disease, cardiac, emergency, and critical‐care settings. A further strength is the deliberate separation of cohort‐level clustering, documented individual‐level coexistence, and temporal sequence, which reduces the risk of interpreting population‐level associations as evidence of within‐patient accumulation or biological interaction. However, important limitations remain. The three stressor domains were defined a priori and incorporated into the search and eligibility framework. Consequently, the synthesis characterizes patterns within these prespecified domains rather than demonstrating that they are the predominant, exhaustive, or independently derived contributors to AKI across East Africa. The review is narrative rather than systematic; no formal study‐level risk‐of‐bias score was applied, and the included studies were heterogeneous in population, AKI definition, baseline creatinine ascertainment, urine‐output measurement, exposure definition, and outcome reporting. More importantly, most studies were not designed to test the multiple‐hit concept directly; they generally examined individual predictors or etiologies and rarely measured exposure combinations, duration, overlap, recovery between events, or statistical interaction at the patient level. The predominance of observational and frequently single‐center studies also leaves treatment‐related findings vulnerable to residual confounding by disease severity and indication. Geographic concentration and limited longitudinal follow‐up further constrain regional generalizability and interpretation of renal recovery. Accordingly, the framework developed here should be interpreted as a structured synthesis of recurrent clinical patterns and evidence gaps, not as proof of a causal, prognostic, or mechanistic multiple‐hit model.

Overall, the current evidence is sufficient to justify broader renal‐risk assessment as illness and treatment evolve, but not to support prognostic weighting of the number or order of renal stressors. The next step is therefore empirical rather than conceptual: Prospective patient‐level studies should determine whether specific combinations, timing, duration, and overlap of exposures add clinically useful information beyond established indicators of disease severity and AKI stage and whether such information changes monitoring or management.

## 5. Conclusion and the Way Forward

The currently available evidence from East Africa indicates that potentially modifiable infectious or inflammatory, hemodynamic, and nephrotoxic contributors recur across diverse clinical settings. However, because the evidence is geographically concentrated, particularly in Ethiopia, Uganda, and Tanzania, these patterns should not be interpreted as representative of all East African settings. Current studies also do not establish how the number, combination, or timing of these stressors influences prognosis. The main contribution of this review is therefore the organization of the available regional evidence into cohort‐level clustering, patient‐level coexistence, and temporal sequence, thereby clarifying what is currently documented and where patient‐level evidence remains insufficient, rather than proposing a new mechanistic model of AKI.

The immediate clinical implication is not to replace disease‐specific assessment with a three‐domain protocol but to broaden renal‐risk evaluation beyond the apparent dominant cause of AKI. Assessment of volume status and hemodynamic compromise, active infection or inflammatory illness, and potentially nephrotoxic exposures may help identify additional modifiable contributors as illness and treatment evolve. Where potentially nephrotoxic therapy is clinically necessary, management should balance therapeutic benefit against renal risk through appropriate dosing, avoidance of additional unnecessary nephrotoxins, and kidney‐function monitoring rather than automatic treatment withdrawal. Reassessment of serum creatinine, urine output, and renal recovery is also important where feasible, particularly in patients with moderate or severe AKI. These measures represent pragmatic clinical priorities derived from the reviewed evidence, not a validated prevention or treatment bundle.

Future research should first determine whether the multiple‐hit perspective has measurable prognostic significance at the individual‐patient level. Prospective multicountry studies should use standardized AKI definitions and record the onset, duration, overlap, and resolution of infection or inflammatory illness, hemodynamic compromise, and nephrotoxic exposure alongside repeated assessment of kidney function. Such designs could establish whether the number, timing, or combination of stressors is independently associated with AKI severity, kidney replacement therapy, renal recovery, mortality, acute kidney disease, or subsequent CKD, while accounting for disease severity and treatment indication. Greater geographic representation is also necessary because the current evidence is concentrated in only six Eastern African countries. If future studies demonstrate that systematic assessment of these domains provides information beyond established clinical evaluation, implementation studies could then test whether a simple three‐domain approach improves recognition, monitoring, or prevention of AKI in resource‐constrained settings.

**Nomenclature**
AHR: Adjusted hazard ratio
AKI: Acute kidney injury
AKIN: Acute Kidney Injury Network
AOR/aOR: Adjusted odds ratio
CI: Confidence interval
CKD: Chronic kidney disease
COVID‐19: Coronavirus disease 2019
HIV: Human immunodeficiency virus
ICU: Intensive care unit
ISN: International Society of Nephrology
KDIGO: Kidney Disease: Improving Global Outcomes
KRT: Kidney replacement therapy
NSAID: Nonsteroidal anti‐inflammatory drug
PCI: Percutaneous coronary intervention
PRISMA: Preferred Reporting Items for Systematic Reviews and Meta‐Analyses
SANRA: Scale for the Assessment of Narrative Review Articles

## Author Contributions

Abdullahi Abdi Yusuf: conceptualization, methodology, and writing–original draft.

Abdihamid Hirsi Abdulle: investigation, writing, and review.

Aden Mohamed Hirsi: investigation, writing, and review.

Ayan Mohamed Hassan: investigation, writing, and review.

Deka Ahmed Abdi: investigation, writing, and review.

Iqra Abdirazak Kabayare: investigation, writing, and review.

Ayan Ahmed Mohomed: investigation, writing, and review.

Khalid Hussein Ulusow: investigation, writing, and review.

## Funding

No funding was received for this work.

## Disclosure

All authors approved the final version of the manuscript.

## Ethics Statement

The authors have nothing to report.

## Consent

The authors have nothing to report.

## Conflicts of Interest

The authors declare no conflicts of interest.

## Supporting Information

Additional supporting information can be found online in the Supporting Information section.

## Supporting information


**Supporting Information** Supporting Table S1. Complete database search strategies for PubMed, Embase, and Scopus, including search dates and numbers of records retrieved.

## Data Availability

Data sharing is not applicable to this article as no datasets were generated or analyzed during the current study.
