# Supplementary material for: Acute Kidney Injury in East Africa in an Era of Converging Renal Stressors: A Multiple‐Hit Narrative Review
Source: Int J Nephrol. 2026 Nov 6;2026:4263572. doi: 10.1155/ijne/4263572 (PMC13743918; doi:10.1155/ijne/4263572)
Supplement: Supplementary file 1 [file IJNE-2026-4263572-s001.docx]

**Supplementary Table S1. Complete database search strategies**

| **Database** | **Search date** | **Records retrieved** |
| --- | --- | --- |
| PubMed | 18 August 2026 | 186 |
| Embase | 18 August 2026 | 384 |
| Scopus | 18 August 2026 | 332 |
| **Total** |  | **902** |

**pubmed**

("Acute Kidney Injury"[Mesh] OR "Kidney Tubular Necrosis, Acute"[Mesh] OR "acute kidney injury"[tiab] OR "acute kidney injuries"[tiab] OR "acute kidney injur*"[tiab] OR "acute renal injury"[tiab] OR "acute renal injuries"[tiab] OR "acute renal injur*"[tiab] OR "acute kidney failure"[tiab] OR "acute renal failure"[tiab] OR "acute kidney insufficien*"[tiab] OR "acute renal insufficien*"[tiab] OR "kidney failure, acute"[tiab] OR "renal failure, acute"[tiab] OR "kidney insufficiency, acute"[tiab] OR "renal insufficiency, acute"[tiab] OR "acute kidney dysfunction"[tiab] OR "acute renal dysfunction"[tiab] OR "acute kidney impairment"[tiab] OR "acute renal impairment"[tiab] OR "acute tubular necrosis"[tiab] OR "acute renal tubular necrosis"[tiab] OR "acute kidney tubular necrosis"[tiab] OR AKI[tiab] OR (ARF[tiab] AND (renal[tiab] OR kidney[tiab])))

AND

("Africa, Eastern"[Mesh:noexp] OR "East Africa"[tiab] OR "Eastern Africa"[tiab] OR "East African"[tiab] OR "Eastern African"[tiab] OR "Horn of Africa"[tiab] OR "African Horn"[tiab] OR "East African Community"[tiab] OR "Burundi"[Mesh] OR Burundi[tiab] OR Burundian*[tiab] OR Bujumbura[tiab] OR Gitega[tiab] OR Ngozi[tiab] OR Rumonge[tiab] OR Muyinga[tiab] OR "Comoros"[Mesh] OR Comoros[tiab] OR Comorian*[tiab] OR Moroni[tiab] OR Mutsamudu[tiab] OR Fomboni[tiab] OR Anjouan[tiab] OR "Grande Comore"[tiab] OR Moheli[tiab] OR "Djibouti"[Mesh] OR Djibouti[tiab] OR Djiboutian*[tiab] OR "Djibouti City"[tiab] OR "Ali Sabieh"[tiab] OR Tadjoura[tiab] OR Tadjourah[tiab] OR Dikhil[tiab] OR Obock[tiab] OR "Eritrea"[Mesh] OR Eritrea[tiab] OR Eritrean*[tiab] OR Asmara[tiab] OR Keren[tiab] OR Massawa[tiab] OR Massaua[tiab] OR Mendefera[tiab] OR Assab[tiab] OR "Ethiopia"[Mesh] OR Ethiopia[tiab] OR Ethiopian*[tiab] OR "Addis Ababa"[tiab] OR Gondar[tiab] OR Gonder[tiab] OR Jimma[tiab] OR Mekelle[tiab] OR Hawassa[tiab] OR Awassa[tiab] OR "Bahir Dar"[tiab] OR "Dire Dawa"[tiab] OR Harar[tiab] OR Adama[tiab] OR Nazret[tiab] OR Dessie[tiab] OR "Arba Minch"[tiab] OR "Kenya"[Mesh] OR Kenya[tiab] OR Kenyan*[tiab] OR Nairobi[tiab] OR Mombasa[tiab] OR Kisumu[tiab] OR Eldoret[tiab] OR Nakuru[tiab] OR Kakamega[tiab] OR Kisii[tiab] OR Nyeri[tiab] OR Meru[tiab] OR Machakos[tiab] OR Kericho[tiab] OR Thika[tiab] OR "Madagascar"[Mesh] OR Madagascar[tiab] OR Malagasy[tiab] OR Antananarivo[tiab] OR Toamasina[tiab] OR Tamatave[tiab] OR Mahajanga[tiab] OR Majunga[tiab] OR Fianarantsoa[tiab] OR Toliara[tiab] OR Tulear[tiab] OR Antsiranana[tiab] OR "Diego Suarez"[tiab] OR "Malawi"[Mesh] OR Malawi[tiab] OR Malawian*[tiab] OR Lilongwe[tiab] OR Blantyre[tiab] OR Zomba[tiab] OR Mzuzu[tiab] OR Mangochi[tiab] OR "Lake Malawi"[tiab] OR "Mauritius"[Mesh] OR Mauritius[tiab] OR Mauritian*[tiab] OR "Port Louis"[tiab] OR Curepipe[tiab] OR "Vacoas-Phoenix"[tiab] OR "Quatre Bornes"[tiab] OR ("Rose Hill"[tiab] AND Mauritius[tiab]) OR "Mozambique"[Mesh] OR Mozambique[tiab] OR Mozambican*[tiab] OR Maputo[tiab] OR Beira[tiab] OR Nampula[tiab] OR Quelimane[tiab] OR Tete[tiab] OR Chimoio[tiab] OR Pemba[tiab] OR "Xai-Xai"[tiab] OR "Rwanda"[Mesh] OR Rwanda[tiab] OR Rwandan*[tiab] OR Kigali[tiab] OR Butare[tiab] OR Huye[tiab] OR Ruhengeri[tiab] OR Musanze[tiab] OR Gisenyi[tiab] OR Rubavu[tiab] OR Kibuye[tiab] OR Karongi[tiab] OR "Seychelles"[Mesh] OR Seychelles[tiab] OR Seychellois[tiab] OR ("Victoria"[tiab] AND Seychelles[tiab]) OR ("Mahe"[tiab] AND Seychelles[tiab]) OR ("Praslin"[tiab] AND Seychelles[tiab]) OR ("La Digue"[tiab] AND Seychelles[tiab]) OR "Somalia"[Mesh] OR Somalia[tiab] OR Somali*[tiab] OR Somaliland[tiab] OR Puntland[tiab] OR Mogadishu[tiab] OR Muqdisho[tiab] OR Hargeisa[tiab] OR Garowe[tiab] OR Garoowe[tiab] OR Bosaso[tiab] OR Bossaso[tiab] OR Boosaaso[tiab] OR Baidoa[tiab] OR Baydhabo[tiab] OR Kismayo[tiab] OR Kismaayo[tiab] OR Galkayo[tiab] OR Gaalkacyo[tiab] OR Beledweyne[tiab] OR Beletweyne[tiab] OR Burao[tiab] OR Burco[tiab] OR "South Sudan"[Mesh] OR "South Sudan"[tiab] OR "South Sudanese"[tiab] OR "Southern Sudan"[tiab] OR Juba[tiab] OR Malakal[tiab] OR ("Wau"[tiab] AND Sudan*[tiab]) OR ("Bor"[tiab] AND "South Sudan"[tiab]) OR Yei[tiab] OR Rumbek[tiab] OR Aweil[tiab] OR "Tanzania"[Mesh] OR Tanzania[tiab] OR Tanzanian*[tiab] OR "United Republic of Tanzania"[tiab] OR Zanzibar[tiab] OR "Dar es Salaam"[tiab] OR Dodoma[tiab] OR Mwanza[tiab] OR Moshi[tiab] OR Kilimanjaro[tiab] OR Arusha[tiab] OR Mbeya[tiab] OR Morogoro[tiab] OR Tanga[tiab] OR Muhimbili[tiab] OR "Uganda"[Mesh] OR Uganda[tiab] OR Ugandan*[tiab] OR Kampala[tiab] OR Mbarara[tiab] OR Gulu[tiab] OR Jinja[tiab] OR Mbale[tiab] OR "Fort Portal"[tiab] OR Lira[tiab] OR Masaka[tiab] OR Entebbe[tiab] OR Soroti[tiab] OR Kabale[tiab] OR Hoima[tiab] OR Mulago[tiab] OR "Zambia"[Mesh] OR Zambia[tiab] OR Zambian*[tiab] OR Lusaka[tiab] OR Ndola[tiab] OR Kitwe[tiab] OR Livingstone[tiab] OR Kabwe[tiab] OR Chipata[tiab] OR Solwezi[tiab] OR ("Mansa"[tiab] AND Zambia[tiab]) OR ("Choma"[tiab] AND Zambia[tiab]) OR "Zimbabwe"[Mesh] OR Zimbabwe[tiab] OR Zimbabwean*[tiab] OR Harare[tiab] OR Bulawayo[tiab] OR Mutare[tiab] OR Gweru[tiab] OR Masvingo[tiab] OR Chitungwiza[tiab])

AND

(("Hypovolemia"[Mesh] OR "Dehydration"[Mesh] OR "Shock"[Mesh] OR "Shock, Hemorrhagic"[Mesh] OR "Hemorrhage"[Mesh] OR "Diarrhea"[Mesh] OR "Vomiting"[Mesh] OR "Gastroenteritis"[Mesh] OR "volume depletion"[tiab] OR "volume depleted"[tiab] OR "intravascular volume depletion"[tiab] OR "intravascular depletion"[tiab] OR "extracellular volume depletion"[tiab] OR "circulating volume depletion"[tiab] OR "effective circulating volume depletion"[tiab] OR hypovolemia[tiab] OR hypovolaemia[tiab] OR hypovolemic[tiab] OR hypovolaemic[tiab] OR dehydration[tiab] OR dehydrated[tiab] OR "fluid depletion"[tiab] OR "fluid loss"[tiab] OR "fluid losses"[tiab] OR "volume loss"[tiab] OR "volume losses"[tiab] OR "reduced circulating volume"[tiab] OR "decreased circulating volume"[tiab] OR "low circulating volume"[tiab] OR "reduced effective circulating volume"[tiab] OR "low effective circulating volume"[tiab] OR "renal hypoperfusion"[tiab] OR "renal hypo-perfusion"[tiab] OR "kidney hypoperfusion"[tiab] OR hypoperfusion[tiab] OR "reduced renal perfusion"[tiab] OR "decreased renal perfusion"[tiab] OR "impaired renal perfusion"[tiab] OR "prerenal acute kidney injury"[tiab] OR "pre-renal acute kidney injury"[tiab] OR "prerenal AKI"[tiab] OR "pre-renal AKI"[tiab] OR "prerenal renal failure"[tiab] OR "pre-renal renal failure"[tiab] OR "prerenal azotemia"[tiab] OR "pre-renal azotemia"[tiab] OR "prerenal azotaemia"[tiab] OR "pre-renal azotaemia"[tiab] OR diarrhea[tiab] OR diarrhoea[tiab] OR diarrheal[tiab] OR diarrhoeal[tiab] OR vomiting[tiab] OR emesis[tiab] OR gastroenteritis[tiab] OR "poor oral intake"[tiab] OR "reduced oral intake"[tiab] OR "decreased oral intake"[tiab] OR "poor fluid intake"[tiab] OR "reduced fluid intake"[tiab] OR "decreased fluid intake"[tiab] OR hemorrhag*[tiab] OR haemorrhag*[tiab] OR bleeding[tiab] OR "blood loss"[tiab] OR "postpartum hemorrhage"[tiab] OR "postpartum haemorrhage"[tiab] OR "obstetric hemorrhage"[tiab] OR "obstetric haemorrhage"[tiab] OR hypotension[tiab] OR hypotensive[tiab] OR shock[tiab] OR "hypovolemic shock"[tiab] OR "hypovolaemic shock"[tiab] OR "hemorrhagic shock"[tiab] OR "haemorrhagic shock"[tiab] OR "circulatory collapse"[tiab] OR "circulatory failure"[tiab] OR burn[tiab] OR burns[tiab] OR "heat stress"[tiab] OR "heat illness"[tiab] OR "heat-related illness"[tiab] OR "heat exposure"[tiab])

OR

("Communicable Diseases"[Mesh] OR "Bacterial Infections"[Mesh] OR "Sepsis"[Mesh] OR "Shock, Septic"[Mesh] OR "Malaria"[Mesh] OR "HIV Infections"[Mesh] OR "Tuberculosis"[Mesh] OR "Leptospirosis"[Mesh] OR "Dengue"[Mesh] OR "Cholera"[Mesh] OR "Gastroenteritis"[Mesh] OR "Pneumonia"[Mesh] OR "Meningitis"[Mesh] OR "Urinary Tract Infections"[Mesh] OR "Pyelonephritis"[Mesh] OR infect*[tiab] OR "infectious disease"[tiab] OR "infectious diseases"[tiab] OR "infection-associated"[tiab] OR "infection associated"[tiab] OR "infection-related"[tiab] OR "infection related"[tiab] OR "infection-associated acute kidney injury"[tiab] OR "infection associated acute kidney injury"[tiab] OR "infection-associated AKI"[tiab] OR sepsis[tiab] OR septic*[tiab] OR "septic shock"[tiab] OR "severe sepsis"[tiab] OR "sepsis-associated acute kidney injury"[tiab] OR "sepsis associated acute kidney injury"[tiab] OR "sepsis-associated AKI"[tiab] OR "sepsis-induced AKI"[tiab] OR "sepsis induced AKI"[tiab] OR bacteremia[tiab] OR bacteraemia[tiab] OR "bloodstream infection"[tiab] OR "bloodstream infections"[tiab] OR "systemic infection"[tiab] OR "systemic infections"[tiab] OR malaria[tiab] OR "severe malaria"[tiab] OR "falciparum malaria"[tiab] OR "cerebral malaria"[tiab] OR Plasmodium[tiab] OR "Plasmodium falciparum"[tiab] OR "malaria-associated acute kidney injury"[tiab] OR "malaria associated acute kidney injury"[tiab] OR "malaria-associated AKI"[tiab] OR HIV[tiab] OR AIDS[tiab] OR "human immunodeficiency virus"[tiab] OR tuberculosis[tiab] OR "Mycobacterium tuberculosis"[tiab] OR leptospirosis[tiab] OR Leptospira[tiab] OR dengue[tiab] OR cholera[tiab] OR "typhoid fever"[tiab] OR "enteric fever"[tiab] OR pneumonia[tiab] OR meningitis[tiab] OR "urinary tract infection"[tiab] OR "urinary tract infections"[tiab] OR pyelonephritis[tiab] OR "diarrheal infection"[tiab] OR "diarrhoeal infection"[tiab] OR "diarrheal disease"[tiab] OR "diarrhoeal disease"[tiab] OR "bacterial infection"[tiab] OR "bacterial infections"[tiab] OR "viral infection"[tiab] OR "viral infections"[tiab] OR "parasitic infection"[tiab] OR "parasitic infections"[tiab] OR "febrile illness"[tiab] OR Ebola[tiab] OR "Ebola virus disease"[tiab] OR Marburg[tiab] OR "Marburg virus disease"[tiab] OR "viral hemorrhagic fever"[tiab] OR "viral haemorrhagic fever"[tiab] OR "yellow fever"[tiab] OR leishmaniasis[tiab] OR "visceral leishmaniasis"[tiab] OR COVID-19[tiab] OR COVID19[tiab] OR SARS-CoV-2[tiab])

OR

("Acute Kidney Injury/chemically induced"[Mesh] OR "Kidney Tubular Necrosis, Acute/chemically induced"[Mesh] OR "Drug-Related Side Effects and Adverse Reactions"[Mesh] OR "Poisoning"[Mesh] OR "Contrast Media"[Mesh] OR "Medicine, Traditional"[Mesh] OR "Medicine, African Traditional"[Mesh] OR "Phytotherapy"[Mesh] OR "Plant Preparations"[Mesh] OR nephrotoxic*[tiab] OR nephrotoxin*[tiab] OR nephrotoxicity[tiab] OR "nephrotoxic exposure"[tiab] OR "nephrotoxic exposures"[tiab] OR "nephrotoxic drug"[tiab] OR "nephrotoxic drugs"[tiab] OR "nephrotoxic medication"[tiab] OR "nephrotoxic medications"[tiab] OR "renal toxicity"[tiab] OR "kidney toxicity"[tiab] OR "toxic nephropathy"[tiab] OR "toxic kidney injury"[tiab] OR "toxic renal injury"[tiab] OR "drug-induced kidney injury"[tiab] OR "drug induced kidney injury"[tiab] OR "drug-induced renal injury"[tiab] OR "drug induced renal injury"[tiab] OR "drug-induced AKI"[tiab] OR "drug induced AKI"[tiab] OR "drug-associated AKI"[tiab] OR "drug associated AKI"[tiab] OR "medication-associated AKI"[tiab] OR "medication associated AKI"[tiab] OR "drug-induced nephrotoxicity"[tiab] OR "drug induced nephrotoxicity"[tiab] OR "medication-induced nephrotoxicity"[tiab] OR aminoglycoside*[tiab] OR gentamicin[tiab] OR amikacin[tiab] OR tobramycin[tiab] OR vancomycin[tiab] OR "amphotericin B"[tiab] OR tenofovir[tiab] OR "tenofovir disoproxil fumarate"[tiab] OR acyclovir[tiab] OR ganciclovir[tiab] OR foscarnet[tiab] OR rifampicin[tiab] OR rifampin[tiab] OR NSAID[tiab] OR NSAIDs[tiab] OR "nonsteroidal anti-inflammatory"[tiab] OR "non-steroidal anti-inflammatory"[tiab] OR ibuprofen[tiab] OR diclofenac[tiab] OR naproxen[tiab] OR "contrast media"[tiab] OR "contrast medium"[tiab] OR "contrast agent"[tiab] OR "contrast agents"[tiab] OR "iodinated contrast"[tiab] OR "radiographic contrast"[tiab] OR radiocontrast[tiab] OR "contrast-associated acute kidney injury"[tiab] OR "contrast associated acute kidney injury"[tiab] OR "contrast-associated AKI"[tiab] OR "contrast associated AKI"[tiab] OR "contrast-induced acute kidney injury"[tiab] OR "contrast induced acute kidney injury"[tiab] OR "contrast-induced AKI"[tiab] OR "contrast induced AKI"[tiab] OR "contrast-induced nephropathy"[tiab] OR "contrast induced nephropathy"[tiab] OR "contrast nephropathy"[tiab] OR CA-AKI[tiab] OR CIN[tiab] OR cisplatin[tiab] OR carboplatin[tiab] OR chemotherapy[tiab] OR "chemotherapy-induced nephrotoxicity"[tiab] OR "chemotherapy induced nephrotoxicity"[tiab] OR methotrexate[tiab] OR cyclosporine[tiab] OR ciclosporin[tiab] OR tacrolimus[tiab] OR "calcineurin inhibitor"[tiab] OR "calcineurin inhibitors"[tiab] OR antiretroviral*[tiab] OR antibiotic*[tiab] OR "traditional medicine"[tiab] OR "traditional medicines"[tiab] OR "traditional African medicine"[tiab] OR "African traditional medicine"[tiab] OR "traditional herbal medicine"[tiab] OR "traditional herbal medicines"[tiab] OR "traditional remedy"[tiab] OR "traditional remedies"[tiab] OR "herbal medicine"[tiab] OR "herbal medicines"[tiab] OR "herbal remedy"[tiab] OR "herbal remedies"[tiab] OR "herbal toxicity"[tiab] OR "herbal nephrotoxicity"[tiab] OR phytotherapy[tiab] OR toxin*[tiab] OR toxic*[tiab] OR poison*[tiab] OR "toxic exposure"[tiab] OR "toxic exposures"[tiab] OR "environmental toxin"[tiab] OR "environmental toxins"[tiab] OR "ethylene glycol"[tiab] OR "diethylene glycol"[tiab] OR pesticide*[tiab] OR herbicide*[tiab] OR "heavy metal"[tiab] OR "heavy metals"[tiab] OR envenomation[tiab] OR envenoming[tiab] OR snakebite[tiab] OR snakebites[tiab] OR "snake bite"[tiab] OR "snake bites"[tiab])

OR

("multiple hit"[tiab] OR "multiple-hit"[tiab] OR "multiple hits"[tiab] OR "multi-hit"[tiab] OR multihit[tiab] OR "two-hit"[tiab] OR "two hit"[tiab] OR "second hit"[tiab] OR "multiple insult"[tiab] OR "multiple insults"[tiab] OR "multiple renal insults"[tiab] OR "multiple kidney insults"[tiab] OR "multiple stressor"[tiab] OR "multiple stressors"[tiab] OR "renal stressor"[tiab] OR "renal stressors"[tiab] OR "kidney stressor"[tiab] OR "kidney stressors"[tiab] OR "concurrent insult"[tiab] OR "concurrent insults"[tiab] OR "concurrent renal insults"[tiab] OR "concurrent kidney insults"[tiab] OR "concurrent exposure"[tiab] OR "concurrent exposures"[tiab] OR "coexisting risk factors"[tiab] OR "co-existing risk factors"[tiab] OR "coexisting exposures"[tiab] OR "co-existing exposures"[tiab] OR "coexisting insults"[tiab] OR "co-existing insults"[tiab] OR "combined exposure"[tiab] OR "combined exposures"[tiab] OR "combined insults"[tiab] OR "overlapping risk factors"[tiab] OR "overlapping exposures"[tiab] OR "overlapping insults"[tiab] OR "overlapping renal insults"[tiab] OR "sequential insult"[tiab] OR "sequential insults"[tiab] OR "sequential exposure"[tiab] OR "sequential exposures"[tiab] OR "cumulative renal stress"[tiab] OR "cumulative kidney stress"[tiab] OR "cumulative renal injury"[tiab] OR "cumulative kidney injury"[tiab] OR "converging stressors"[tiab] OR "convergent stressors"[tiab] OR "convergence of stressors"[tiab] OR "convergence of renal stressors"[tiab] OR "convergence of kidney stressors"[tiab] OR multifactorial[tiab] OR "multifactorial acute kidney injury"[tiab] OR "multifactorial AKI"[tiab] OR "multiple etiologies"[tiab] OR "multiple aetiologies"[tiab] OR "mixed etiology"[tiab] OR "mixed aetiology"[tiab]))

AND

("2012/01/01"[Date - Publication] : "2026/08/18"[Date - Publication])

**Embase**

('acute kidney injury'/exp OR ('acute kidney injury' OR 'acute kidney injuries' OR 'acute kidney injur*' OR 'acute renal injury' OR 'acute renal injuries' OR 'acute renal injur*' OR 'acute kidney failure' OR 'acute renal failure' OR 'acute kidney insufficien*' OR 'acute renal insufficien*' OR 'kidney failure, acute' OR 'renal failure, acute' OR 'kidney insufficiency, acute' OR 'renal insufficiency, acute' OR 'acute kidney dysfunction' OR 'acute renal dysfunction' OR 'acute kidney impairment' OR 'acute renal impairment' OR 'acute tubular necrosis' OR 'acute renal tubular necrosis' OR 'acute kidney tubular necrosis' OR AKI OR (ARF AND (renal OR kidney))):ti,ab,kw)

AND

(('East Africa' OR 'Eastern Africa' OR 'East African' OR 'Eastern African' OR 'Horn of Africa' OR 'African Horn' OR 'East African Community' OR Burundi OR Burundian* OR Bujumbura OR Gitega OR Ngozi OR Rumonge OR Muyinga OR Comoros OR Comorian* OR Moroni OR Mutsamudu OR Fomboni OR Anjouan OR 'Grande Comore' OR Moheli OR Djibouti OR Djiboutian* OR 'Djibouti City' OR 'Ali Sabieh' OR Tadjoura OR Tadjourah OR Dikhil OR Obock OR Eritrea OR Eritrean* OR Asmara OR Keren OR Massawa OR Massaua OR Mendefera OR Assab OR Ethiopia OR Ethiopian* OR 'Addis Ababa' OR Gondar OR Gonder OR Jimma OR Mekelle OR Hawassa OR Awassa OR 'Bahir Dar' OR 'Dire Dawa' OR Harar OR Adama OR Nazret OR Dessie OR 'Arba Minch' OR Kenya OR Kenyan* OR Nairobi OR Mombasa OR Kisumu OR Eldoret OR Nakuru OR Kakamega OR Kisii OR Nyeri OR Meru OR Machakos OR Kericho OR Thika OR Madagascar OR Malagasy OR Antananarivo OR Toamasina OR Tamatave OR Mahajanga OR Majunga OR Fianarantsoa OR Toliara OR Tulear OR Antsiranana OR 'Diego Suarez' OR Malawi OR Malawian* OR Lilongwe OR Blantyre OR Zomba OR Mzuzu OR Mangochi OR 'Lake Malawi' OR Mauritius OR Mauritian* OR 'Port Louis' OR Curepipe OR 'Vacoas-Phoenix' OR 'Quatre Bornes' OR ('Rose Hill' AND Mauritius) OR Mozambique OR Mozambican* OR Maputo OR Beira OR Nampula OR Quelimane OR Tete OR Chimoio OR Pemba OR 'Xai-Xai' OR Rwanda OR Rwandan* OR Kigali OR Butare OR Huye OR Ruhengeri OR Musanze OR Gisenyi OR Rubavu OR Kibuye OR Karongi OR Seychelles OR Seychellois OR (Victoria AND Seychelles) OR (Mahe AND Seychelles) OR (Praslin AND Seychelles) OR ('La Digue' AND Seychelles) OR Somalia OR Somali* OR Somaliland OR Puntland OR Mogadishu OR Muqdisho OR Hargeisa OR Garowe OR Garoowe OR Bosaso OR Bossaso OR Boosaaso OR Baidoa OR Baydhabo OR Kismayo OR Kismaayo OR Galkayo OR Gaalkacyo OR Beledweyne OR Beletweyne OR Burao OR Burco OR 'South Sudan' OR 'South Sudanese' OR 'Southern Sudan' OR Juba OR Malakal OR (Wau AND Sudan*) OR (Bor AND 'South Sudan') OR Yei OR Rumbek OR Aweil OR Tanzania OR Tanzanian* OR 'United Republic of Tanzania' OR Zanzibar OR 'Dar es Salaam' OR Dodoma OR Mwanza OR Moshi OR Kilimanjaro OR Arusha OR Mbeya OR Morogoro OR Tanga OR Muhimbili OR Uganda OR Ugandan* OR Kampala OR Mbarara OR Gulu OR Jinja OR Mbale OR 'Fort Portal' OR Lira OR Masaka OR Entebbe OR Soroti OR Kabale OR Hoima OR Mulago OR Zambia OR Zambian* OR Lusaka OR Ndola OR Kitwe OR Livingstone OR Kabwe OR Chipata OR Solwezi OR (Mansa AND Zambia) OR (Choma AND Zambia) OR Zimbabwe OR Zimbabwean* OR Harare OR Bulawayo OR Mutare OR Gweru OR Masvingo OR Chitungwiza):ti,ab,kw)

AND

(('volume depletion' OR 'volume depleted' OR 'intravascular volume depletion' OR 'intravascular depletion' OR 'extracellular volume depletion' OR 'circulating volume depletion' OR 'effective circulating volume depletion' OR hypovolemia OR hypovolaemia OR hypovolemic OR hypovolaemic OR dehydration OR dehydrated OR 'fluid depletion' OR 'fluid loss' OR 'fluid losses' OR 'volume loss' OR 'volume losses' OR 'reduced circulating volume' OR 'decreased circulating volume' OR 'low circulating volume' OR 'reduced effective circulating volume' OR 'low effective circulating volume' OR 'renal hypoperfusion' OR 'renal hypo-perfusion' OR 'kidney hypoperfusion' OR hypoperfusion OR 'reduced renal perfusion' OR 'decreased renal perfusion' OR 'impaired renal perfusion' OR 'prerenal acute kidney injury' OR 'pre-renal acute kidney injury' OR 'prerenal AKI' OR 'pre-renal AKI' OR 'prerenal renal failure' OR 'pre-renal renal failure' OR 'prerenal azotemia' OR 'pre-renal azotemia' OR 'prerenal azotaemia' OR 'pre-renal azotaemia' OR diarrhea OR diarrhoea OR diarrheal OR diarrhoeal OR vomiting OR emesis OR gastroenteritis OR 'poor oral intake' OR 'reduced oral intake' OR 'decreased oral intake' OR 'poor fluid intake' OR 'reduced fluid intake' OR 'decreased fluid intake' OR hemorrhag* OR haemorrhag* OR bleeding OR 'blood loss' OR 'postpartum hemorrhage' OR 'postpartum haemorrhage' OR 'obstetric hemorrhage' OR 'obstetric haemorrhage' OR hypotension OR hypotensive OR shock OR 'hypovolemic shock' OR 'hypovolaemic shock' OR 'hemorrhagic shock' OR 'haemorrhagic shock' OR 'circulatory collapse' OR 'circulatory failure' OR burn OR burns OR 'heat stress' OR 'heat illness' OR 'heat-related illness' OR 'heat exposure'):ti,ab,kw

OR

(infection OR infections OR infectious OR infect* OR 'infectious disease' OR 'infectious diseases' OR 'infection-associated' OR 'infection associated' OR 'infection-related' OR 'infection related' OR 'infection-associated acute kidney injury' OR 'infection associated acute kidney injury' OR 'infection-associated AKI' OR sepsis OR septic* OR 'septic shock' OR 'severe sepsis' OR 'sepsis-associated acute kidney injury' OR 'sepsis associated acute kidney injury' OR 'sepsis-associated AKI' OR 'sepsis-induced AKI' OR 'sepsis induced AKI' OR bacteremia OR bacteraemia OR 'bloodstream infection' OR 'bloodstream infections' OR 'systemic infection' OR 'systemic infections' OR malaria OR 'severe malaria' OR 'falciparum malaria' OR 'cerebral malaria' OR Plasmodium OR 'Plasmodium falciparum' OR 'malaria-associated acute kidney injury' OR 'malaria associated acute kidney injury' OR 'malaria-associated AKI' OR HIV OR AIDS OR 'human immunodeficiency virus' OR tuberculosis OR 'Mycobacterium tuberculosis' OR leptospirosis OR Leptospira OR dengue OR cholera OR 'typhoid fever' OR 'enteric fever' OR pneumonia OR meningitis OR 'urinary tract infection' OR 'urinary tract infections' OR pyelonephritis OR 'diarrheal infection' OR 'diarrhoeal infection' OR 'diarrheal disease' OR 'diarrhoeal disease' OR 'bacterial infection' OR 'bacterial infections' OR 'viral infection' OR 'viral infections' OR 'parasitic infection' OR 'parasitic infections' OR 'febrile illness' OR Ebola OR 'Ebola virus disease' OR Marburg OR 'Marburg virus disease' OR 'viral hemorrhagic fever' OR 'viral haemorrhagic fever' OR 'yellow fever' OR leishmaniasis OR 'visceral leishmaniasis' OR 'COVID-19' OR COVID19 OR 'SARS-CoV-2'):ti,ab,kw

OR

(nephrotoxic* OR nephrotoxin* OR nephrotoxicity OR 'nephrotoxic exposure' OR 'nephrotoxic exposures' OR 'nephrotoxic drug' OR 'nephrotoxic drugs' OR 'nephrotoxic medication' OR 'nephrotoxic medications' OR 'renal toxicity' OR 'kidney toxicity' OR 'toxic nephropathy' OR 'toxic kidney injury' OR 'toxic renal injury' OR 'drug-induced kidney injury' OR 'drug induced kidney injury' OR 'drug-induced renal injury' OR 'drug induced renal injury' OR 'drug-induced AKI' OR 'drug induced AKI' OR 'drug-associated AKI' OR 'drug associated AKI' OR 'medication-associated AKI' OR 'medication associated AKI' OR 'drug-induced nephrotoxicity' OR 'drug induced nephrotoxicity' OR 'medication-induced nephrotoxicity' OR 'drug toxicity' OR 'drug toxicities' OR aminoglycoside* OR gentamicin OR amikacin OR tobramycin OR vancomycin OR 'amphotericin B' OR tenofovir OR 'tenofovir disoproxil fumarate' OR acyclovir OR ganciclovir OR foscarnet OR rifampicin OR rifampin OR NSAID OR NSAIDs OR 'nonsteroidal anti-inflammatory' OR 'non-steroidal anti-inflammatory' OR ibuprofen OR diclofenac OR naproxen OR 'analgesic nephropathy' OR 'contrast media' OR 'contrast medium' OR 'contrast agent' OR 'contrast agents' OR 'iodinated contrast' OR 'radiographic contrast' OR radiocontrast OR 'contrast-associated acute kidney injury' OR 'contrast associated acute kidney injury' OR 'contrast-associated AKI' OR 'contrast associated AKI' OR 'contrast-induced acute kidney injury' OR 'contrast induced acute kidney injury' OR 'contrast-induced AKI' OR 'contrast induced AKI' OR 'contrast-induced nephropathy' OR 'contrast induced nephropathy' OR 'contrast nephropathy' OR 'CA-AKI' OR CIN OR cisplatin OR carboplatin OR chemotherapy OR 'chemotherapy-induced nephrotoxicity' OR 'chemotherapy induced nephrotoxicity' OR methotrexate OR cyclosporine OR ciclosporin OR tacrolimus OR 'calcineurin inhibitor' OR 'calcineurin inhibitors' OR antiretroviral* OR antibiotic* OR 'traditional medicine' OR 'traditional medicines' OR 'traditional African medicine' OR 'African traditional medicine' OR 'traditional herbal medicine' OR 'traditional herbal medicines' OR 'traditional remedy' OR 'traditional remedies' OR 'herbal medicine' OR 'herbal medicines' OR 'herbal remedy' OR 'herbal remedies' OR 'herbal toxicity' OR 'herbal nephrotoxicity' OR phytotherapy OR toxin* OR toxic* OR poison* OR 'toxic exposure' OR 'toxic exposures' OR 'environmental toxin' OR 'environmental toxins' OR 'ethylene glycol' OR 'diethylene glycol' OR pesticide* OR herbicide* OR 'heavy metal' OR 'heavy metals' OR envenomation OR envenoming OR snakebite OR snakebites OR 'snake bite' OR 'snake bites'):ti,ab,kw

OR

('multiple hit' OR 'multiple-hit' OR 'multiple hits' OR 'multi-hit' OR multihit OR 'two-hit' OR 'two hit' OR 'second hit' OR 'multiple insult' OR 'multiple insults' OR 'multiple renal insults' OR 'multiple kidney insults' OR 'multiple stressor' OR 'multiple stressors' OR 'renal stressor' OR 'renal stressors' OR 'kidney stressor' OR 'kidney stressors' OR 'concurrent insult' OR 'concurrent insults' OR 'concurrent renal insults' OR 'concurrent kidney insults' OR 'concurrent exposure' OR 'concurrent exposures' OR 'coexisting risk factors' OR 'co-existing risk factors' OR 'coexisting exposures' OR 'co-existing exposures' OR 'coexisting insults' OR 'co-existing insults' OR 'combined exposure' OR 'combined exposures' OR 'combined insults' OR 'overlapping risk factors' OR 'overlapping exposures' OR 'overlapping insults' OR 'overlapping renal insults' OR 'sequential insult' OR 'sequential insults' OR 'sequential exposure' OR 'sequential exposures' OR 'cumulative renal stress' OR 'cumulative kidney stress' OR 'cumulative renal injury' OR 'cumulative kidney injury' OR 'converging stressors' OR 'convergent stressors' OR 'convergence of stressors' OR 'convergence of renal stressors' OR 'convergence of kidney stressors' OR multifactorial OR 'multifactorial acute kidney injury' OR 'multifactorial AKI' OR 'multiple etiologies' OR 'multiple aetiologies' OR 'mixed etiology' OR 'mixed aetiology'):ti,ab,kw)

AND [2012-2026]/py

**Scopus**

TITLE-ABS-KEY("acute kidney injury" OR "acute renal injury" OR "acute kidney failure" OR "acute renal failure" OR "acute kidney insufficien*" OR "acute renal insufficien*" OR "acute kidney dysfunction" OR "acute renal dysfunction" OR "acute kidney impairment" OR "acute renal impairment" OR "acute tubular necrosis" OR "acute renal tubular necrosis" OR "acute kidney tubular necrosis" OR AKI OR (ARF AND (renal OR kidney)))

AND

TITLE-ABS-KEY("East Africa" OR "Eastern Africa" OR "East African" OR "Eastern African" OR "Horn of Africa" OR "African Horn" OR "East African Community" OR Burundi OR Burundian* OR Bujumbura OR Gitega OR Ngozi OR Rumonge OR Muyinga OR Comoros OR Comorian* OR Moroni OR Mutsamudu OR Fomboni OR Anjouan OR "Grande Comore" OR Moheli OR Djibouti OR Djiboutian* OR "Djibouti City" OR "Ali Sabieh" OR Tadjoura OR Tadjourah OR Dikhil OR Obock OR Eritrea OR Eritrean* OR Asmara OR Keren OR Massawa OR Massaua OR Mendefera OR Assab OR Ethiopia OR Ethiopian* OR "Addis Ababa" OR Gondar OR Gonder OR Jimma OR Mekelle OR Hawassa OR Awassa OR "Bahir Dar" OR "Dire Dawa" OR Harar OR Adama OR Nazret OR Dessie OR "Arba Minch" OR Kenya OR Kenyan* OR Nairobi OR Mombasa OR Kisumu OR Eldoret OR Nakuru OR Kakamega OR Kisii OR Nyeri OR Meru OR Machakos OR Kericho OR Thika OR Madagascar OR Malagasy OR Antananarivo OR Toamasina OR Tamatave OR Mahajanga OR Majunga OR Fianarantsoa OR Toliara OR Tulear OR Antsiranana OR "Diego Suarez" OR Malawi OR Malawian* OR Lilongwe OR Blantyre OR Zomba OR Mzuzu OR Mangochi OR "Lake Malawi" OR Mauritius OR Mauritian* OR "Port Louis" OR Curepipe OR "Vacoas-Phoenix" OR "Quatre Bornes" OR ("Rose Hill" AND Mauritius) OR Mozambique OR Mozambican* OR Maputo OR Beira OR Nampula OR Quelimane OR Tete OR Chimoio OR Pemba OR "Xai-Xai" OR Rwanda OR Rwandan* OR Kigali OR Butare OR Huye OR Ruhengeri OR Musanze OR Gisenyi OR Rubavu OR Kibuye OR Karongi OR Seychelles OR Seychellois OR (Victoria AND Seychelles) OR (Mahe AND Seychelles) OR (Praslin AND Seychelles) OR ("La Digue" AND Seychelles) OR Somalia OR Somali* OR Somaliland OR Puntland OR Mogadishu OR Muqdisho OR Hargeisa OR Garowe OR Garoowe OR Bosaso OR Bossaso OR Boosaaso OR Baidoa OR Baydhabo OR Kismayo OR Kismaayo OR Galkayo OR Gaalkacyo OR Beledweyne OR Beletweyne OR Burao OR Burco OR "South Sudan" OR "South Sudanese" OR "Southern Sudan" OR Juba OR Malakal OR (Wau AND Sudan*) OR (Bor AND "South Sudan") OR Yei OR Rumbek OR Aweil OR Tanzania OR Tanzanian* OR "United Republic of Tanzania" OR Zanzibar OR "Dar es Salaam" OR Dodoma OR Mwanza OR Moshi OR Kilimanjaro OR Arusha OR Mbeya OR Morogoro OR Tanga OR Muhimbili OR Uganda OR Ugandan* OR Kampala OR Mbarara OR Gulu OR Jinja OR Mbale OR "Fort Portal" OR Lira OR Masaka OR Entebbe OR Soroti OR Kabale OR Hoima OR Mulago OR Zambia OR Zambian* OR Lusaka OR Ndola OR Kitwe OR Livingstone OR Kabwe OR Chipata OR Solwezi OR (Mansa AND Zambia) OR (Choma AND Zambia) OR Zimbabwe OR Zimbabwean* OR Harare OR Bulawayo OR Mutare OR Gweru OR Masvingo OR Chitungwiza)

AND

TITLE-ABS-KEY(

("volume depletion" OR "volume depleted" OR "intravascular volume depletion" OR "intravascular depletion" OR "extracellular volume depletion" OR "circulating volume depletion" OR "effective circulating volume depletion" OR hypovolemia OR hypovolaemia OR hypovolemic OR hypovolaemic OR dehydration OR dehydrated OR "fluid depletion" OR "fluid loss" OR "volume loss" OR "reduced circulating volume" OR "decreased circulating volume" OR "low circulating volume" OR "reduced effective circulating volume" OR "renal hypoperfusion" OR "renal hypo-perfusion" OR "kidney hypoperfusion" OR hypoperfusion OR "reduced renal perfusion" OR "decreased renal perfusion" OR "impaired renal perfusion" OR "prerenal acute kidney injury" OR "pre-renal acute kidney injury" OR "prerenal AKI" OR "pre-renal AKI" OR "prerenal renal failure" OR "pre-renal renal failure" OR "prerenal azotemia" OR "pre-renal azotemia" OR "prerenal azotaemia" OR "pre-renal azotaemia" OR diarrhea OR diarrhoea OR diarrheal OR diarrhoeal OR vomiting OR emesis OR gastroenteritis OR "poor oral intake" OR "reduced oral intake" OR "decreased oral intake" OR "poor fluid intake" OR "reduced fluid intake" OR "decreased fluid intake" OR hemorrhag* OR haemorrhag* OR bleeding OR "blood loss" OR "postpartum hemorrhage" OR "postpartum haemorrhage" OR "obstetric hemorrhage" OR "obstetric haemorrhage" OR hypotension OR hypotensive OR shock OR "hypovolemic shock" OR "hypovolaemic shock" OR "hemorrhagic shock" OR "haemorrhagic shock" OR "circulatory collapse" OR "circulatory failure" OR burn OR burns OR "heat stress" OR "heat illness" OR "heat-related illness" OR "heat exposure")

OR

(infection OR infections OR infectious OR infect* OR "infectious disease" OR "infection-associated" OR "infection associated" OR "infection-related" OR "infection related" OR "infection-associated acute kidney injury" OR "infection-associated AKI" OR sepsis OR septic* OR "septic shock" OR "severe sepsis" OR "sepsis-associated acute kidney injury" OR "sepsis associated acute kidney injury" OR "sepsis-associated AKI" OR "sepsis-induced AKI" OR "sepsis induced AKI" OR bacteremia OR bacteraemia OR "bloodstream infection" OR "systemic infection" OR malaria OR "severe malaria" OR "falciparum malaria" OR "cerebral malaria" OR Plasmodium OR "Plasmodium falciparum" OR "malaria-associated acute kidney injury" OR "malaria-associated AKI" OR HIV OR AIDS OR "human immunodeficiency virus" OR tuberculosis OR "Mycobacterium tuberculosis" OR leptospirosis OR Leptospira OR dengue OR cholera OR "typhoid fever" OR "enteric fever" OR pneumonia OR meningitis OR "urinary tract infection" OR pyelonephritis OR "diarrheal infection" OR "diarrhoeal infection" OR "diarrheal disease" OR "diarrhoeal disease" OR "bacterial infection" OR "viral infection" OR "parasitic infection" OR "febrile illness" OR Ebola OR "Ebola virus disease" OR Marburg OR "Marburg virus disease" OR "viral hemorrhagic fever" OR "viral haemorrhagic fever" OR "yellow fever" OR leishmaniasis OR "visceral leishmaniasis" OR "COVID-19" OR COVID19 OR "SARS-CoV-2")

OR

(nephrotoxic* OR nephrotoxin* OR nephrotoxicity OR "nephrotoxic exposure" OR "nephrotoxic drug" OR "nephrotoxic medication" OR "renal toxicity" OR "kidney toxicity" OR "toxic nephropathy" OR "toxic kidney injury" OR "toxic renal injury" OR "drug-induced kidney injury" OR "drug induced kidney injury" OR "drug-induced renal injury" OR "drug induced renal injury" OR "drug-induced AKI" OR "drug induced AKI" OR "drug-associated AKI" OR "drug associated AKI" OR "medication-associated AKI" OR "medication associated AKI" OR "drug-induced nephrotoxicity" OR "drug induced nephrotoxicity" OR "medication-induced nephrotoxicity" OR "drug toxicity" OR aminoglycoside* OR gentamicin OR amikacin OR tobramycin OR vancomycin OR "amphotericin B" OR tenofovir OR "tenofovir disoproxil fumarate" OR acyclovir OR ganciclovir OR foscarnet OR rifampicin OR rifampin OR NSAID OR NSAIDs OR "nonsteroidal anti-inflammatory" OR "non-steroidal anti-inflammatory" OR ibuprofen OR diclofenac OR naproxen OR "analgesic nephropathy" OR "contrast media" OR "contrast medium" OR "contrast agent" OR "iodinated contrast" OR "radiographic contrast" OR radiocontrast OR "contrast-associated acute kidney injury" OR "contrast associated acute kidney injury" OR "contrast-associated AKI" OR "contrast-induced acute kidney injury" OR "contrast induced acute kidney injury" OR "contrast-induced AKI" OR "contrast-induced nephropathy" OR "contrast induced nephropathy" OR "contrast nephropathy" OR "CA-AKI" OR CIN OR cisplatin OR carboplatin OR chemotherapy OR "chemotherapy-induced nephrotoxicity" OR methotrexate OR cyclosporine OR ciclosporin OR tacrolimus OR "calcineurin inhibitor" OR antiretroviral* OR antibiotic* OR "traditional medicine" OR "traditional medicines" OR "traditional African medicine" OR "African traditional medicine" OR "traditional herbal medicine" OR "traditional herbal medicines" OR "traditional remedy" OR "traditional remedies" OR "herbal medicine" OR "herbal medicines" OR "herbal remedy" OR "herbal remedies" OR "herbal toxicity" OR "herbal nephrotoxicity" OR phytotherapy OR toxin* OR toxic* OR poison* OR "toxic exposure" OR "environmental toxin" OR "ethylene glycol" OR "diethylene glycol" OR pesticide* OR herbicide* OR "heavy metal" OR "heavy metals" OR envenomation OR envenoming OR snakebite OR snakebites OR "snake bite")

OR

("multiple hit" OR "multiple-hit" OR "multiple hits" OR "multi-hit" OR multihit OR "two-hit" OR "two hit" OR "second hit" OR "multiple insult" OR "multiple insults" OR "multiple renal insults" OR "multiple kidney insults" OR "multiple stressor" OR "multiple stressors" OR "renal stressor" OR "renal stressors" OR "kidney stressor" OR "kidney stressors" OR "concurrent insult" OR "concurrent insults" OR "concurrent renal insults" OR "concurrent kidney insults" OR "concurrent exposure" OR "concurrent exposures" OR "coexisting risk factors" OR "co-existing risk factors" OR "coexisting exposures" OR "co-existing exposures" OR "coexisting insults" OR "co-existing insults" OR "combined exposure" OR "combined exposures" OR "combined insults" OR "overlapping risk factors" OR "overlapping exposures" OR "overlapping insults" OR "overlapping renal insults" OR "sequential insult" OR "sequential insults" OR "sequential exposure" OR "sequential exposures" OR "cumulative renal stress" OR "cumulative kidney stress" OR "cumulative renal injury" OR "cumulative kidney injury" OR "converging stressors" OR "convergent stressors" OR "convergence of stressors" OR "convergence of renal stressors" OR "convergence of kidney stressors" OR multifactorial OR "multifactorial acute kidney injury" OR "multifactorial AKI" OR "multiple etiologies" OR "multiple aetiologies" OR "mixed etiology" OR "mixed aetiology")

)

AND PUBYEAR > 2011

AND PUBYEAR < 2027
